# PLHI-MC10: A dataset of exercise activities captured through a triple synchronous medically-approved sensor

**DOI:** 10.1016/j.dib.2021.107287

**Published:** 2021-08-20

**Authors:** Yohan Mahajan, Ananth Bhimireddy, Areeba Abid, Judy W. Gichoya, Saptarshi Purkayastha

**Affiliations:** a Indiana University Purdue University Indianapolis, Indianapolis, IN 46202, USA; b Emory University School of Medicine, 100 Woodruff Circle, Atlanta, GA 30322, USA

**Keywords:** Human activity recognition, Accelerometer, Gyroscope, Electrode values

## Abstract

Most human activity recognition datasets that are publicly available have data captured by using either smartphones or smartwatches, which are usually placed on the waist or the wrist, respectively. These devices obtain one set of acceleration and angular velocity in the *x*-, *y*-, and *z*-axis from the accelerometer and the gyroscope planted in these devices. The PLHI-MC10 dataset contains data obtained by using 3 BioStamp nPoint® sensors from 7 physically healthy adult test subjects performing different exercise activities. These sensors are the state-of-the-art biomedical sensors manufactured by MC10. Each of the three sensors was attached to the subject externally on three muscles-Extensor Digitorum (Posterior Forearm), Gastrocnemius (Calf), and Pectoralis (Chest)-giving us three sets of 3 axial acceleration, two sets of 3 axial angular velocities, and 1 set of voltage values from the heart. Using three different sensors instead of a single sensor improves precision. It helps distinguish between human activities as it simultaneously captures the movement and contractions of various muscles from separate parts of the human body. Each test subject performed five activities (stairs, jogging, skipping, lifting kettlebell, basketball throws) in a supervised environment. The data is cleaned, filtered, and synced.

## Specifications Table

**Table:** 

Subject	Health and medical sciences: Sports science, therapy and medicine.
Specific subject area	Human Activity Recognition
Type of data	Table
How data were acquired	Instruments: Wearable sensors (accelerometer, gyroscope, and electrode values). Make and model of the instruments used: MC10 Inc. BioStamp nPoint® sensors.
Data format	Raw Analyzed Filtered
Parameters for data collection	The frequency of data collection for accelerometer and gyroscope embedded in the Extensor Digitorum and Gastrocnemius sensors is 62.5 Hz, whereas the frequency of data collection for accelerometer and electrode embedded in the Pectoralis sensor is 31.25 Hz and 250 Hz, respectively.
Description of data collection	Each of the 7 subjects performed all 5 activities in a supervised environment while wearing the biomedical activity sensors. The exact start and end times of each activity were accurately recorded.
Data source location	Research lab: Purkayastha Lab for Health Innovation (PLHI). Institution: Indiana University-Purdue University Indianapolis. City/Town/Region: Indianapolis. Country: United States of America.
Data accessibility	Repository name: GitHub Data URL: https://github.com/iupui-soic/plhi-mc10

## Value of the Data

• This data contains synchronous motion and muscle measurements at 3 points on the body (back of the forearm, upper calf muscle, chest) for a range of body types. This heterogeneous data is useful for the classification and recognition of various activities [1].
• The data helps detect and/or classify daily activities and sports (*e.g*., detecting jogging, counting free throws, etc.) from wearable sensors. Researchers may also use it to validate algorithms (classification, clustering, or others) [2].
• The time-series data can be analyzed and further processed to extract identifying features of various activities. Additionally, it can be used to develop and validate classification algorithms for activity recognition [3].
• Educators may use this dataset to train students in data mining or machine learning.

## 1 Data Description

BioStamp nPoint® is an FDA 510(k) cleared medical device designed to collect medical-grade, clinical quality biometric, physiological, and electronic clinical outcomes assessment (eCOA) data [4]. Data collection was approved by Indiana University's Institutional Review Board (IRB: #2010321996).

### 1.1 Raw data

The raw data directory consists of 7 sub-directories. Each one of these sub-directories consists of data collected from each subject. Every subject has 3 folders, each corresponding to one of the three BioStamp nPoint® sensors attached to the subject's body. These folders are named according to the muscles where the sensor was attached - Extensor Digitorum (posterior forearm), Gastrocnemius (calf), and Pectoralis (chest).

Both Extensor Digitorum and Gastrocnemius sensors consist of embedded accelerometer and gyroscope whose reading is stored in two different files - *accel.csv* and *gyro.csv*, respectively. The frequency of data collection for both, accelerometer and gyroscope is 62.5 Hz and the data collected is along all the 3 axes.

The Pectoralis sensor captures accelerometer and electrode value readings. Like the Extensor Digitorum and Gastrocnemius sensors, accelerometer readings are collected along the 3 axes, as shown in Table 1. The accelerometer readings can be found in the file *accel.csv*, whereas electrode values are in *elec.csv*. The frequency of data collection of the accelerometer and electrode values is 31.25 Hz and 250 Hz, respectively. Each data point collected by each sensor is associated with a timestamp, indicating the exact time in microseconds at which the particular data point was generated.

**Table 1:** Accelerometer.

Attributes	Data Type	Description
Timestamp (microseconds)	Timestamp	Time in GMT Unix Epoch microseconds
Accel X (g)	Float	Acceleration values in G's
Accel Y (g)	Float	Acceleration values in G's
Accel Z (g)	Float	Acceleration values in G's

**Table 2:** Gyroscope.

Attributes	Data Type	Description
Timestamp (microseconds)	Timestamp	Time in GMT Unix Epoch microseconds
Gyro X (Â °/s)	Float	Gyroscope values in degrees per second
Gyro Y (Â °/s)	Float	Gyroscope values in degrees per second
Gyro Z (Â °/s)	Float	Gyroscope values in degrees per second

**Table 3:** Electrode values.

Attributes	Data Type	Description
Timestamp (microseconds)	Timestamp	Time in GMT Unix Epoch microseconds
Sample (V)	Float	Voltages values in Volts

**Table 4:** Metadata.

Attributes	Data Type	Description
Activity	String	Activity Label
Starting Time	Timestamp	The start time of activity in EST
Elapsed Time	Integer	The time taken to complete each activity in seconds

### 1.2 Metadata

As every subject performed a set of five predetermined activities, the exact start time and the time taken to complete each activity were recorded. This information helped in labeling every data point with one of the five activity labels. Since the number of subjects for this experiment was seven, there are seven metadata files, one for each subject.

### 1.3 Demographic data

Table 5 shows description and structure of the demographic data.

**Table 5:** Demographic data.

Attributes	Data Type	Description
Subject ID	Integer	Subject identifier
Gender	String	Gender of the subject
Age	Integer	Age of the subject in years
Weight	Integer	Weight of the subject in pounds (lbs)
Height	String	Height of the subject in feet+inches

### 1.4 Filtered data

A filtered table (Table 6) is constructed by adding activity labels to each table (*accel.csv, gyro.csv*, and *elec.csv*) from each sensor, for every subject with the help of metadata tables provided for each subject. The readings from all the sensors are then merged. The merged data from all the subjects are then concatenated to get the final filtered table.

**Table 6:** Filtered data.

Attributes	Data Type
Subject ID	Integer
Timestamp (microseconds)	Timestamp
Accel X (g) leg	Float
Accel Y (g) leg	Float
Accel Z (g) leg	Float
Gyro X (Â °/s) leg	Float
Gyro Y (Â °/s) leg	Float
Gyro Z (Â °/s) leg	Float
Accel X (g) hand	Float
Accel Y (g) hand	Float
Accel Z (g) hand	Float
Gyro X (Â °/s) hand	Float
Gyro Y (Â °/s) hand	Float
Gyro Z (Â °/s) hand	Float
Accel X (g) chest	Float
Accel Y (g) chest	Float
Accel Z (g) chest	Float
Sample (V)	Float
Activity Label	String

The final filtered table has more than 142 K data points with 19 attributes. The data in the filtered table is un-normalized and un-standardized.

### 1.5 Data statistics

Data Statistics table provides the descriptive statistical information of the entire filtered dataset. It gives us the count of data points, mean, standard deviation, minimum value, 25th percentile, 50th percentile, 75th percentile and the maximum value for each activity, captured by each sensor. Table 7 depicts the subset of the actual data statistics, which can be found on the repository, which illustrates all the above mentioned statistical features across all the five activities, captured by the Accel X (g) leg sensor.

**Table 7:** Data statistics.

Sensor	Statistical Atrribute	Basket Ball	Jog	Kettle Bell	Skippings	Stairs
Accel X (g) leg	Count	28218	37763	18445	9003	48644
Accel X (g) leg	Mean	0.70	0.50	0.78	0.56	0.53
Accel X (g) leg	STD	0.67	1.32	0.58	1.60	1.20
Accel X (g) leg	Min	-1.34	-4.00	-1.48	-4.00	-4.00
Accel X (g) leg	25%	0.92	-0.41	0.92	-0.33	-0.34
Accel X (g) leg	50%	0.98	0.75	0.98	0.82	0.82
Accel X (g) leg	75%	0.99	1.29	0.99	1.26	1.22
Accel X (g) leg	Max	4.00	4.00	2.64	4.00	4.00

**Table 8:** Activities.

Activity	Instructions
Stairs	Climb up and down two flights of stairs, two times.
Jogging	Jog for 150 m
Skipping	20 rounds of skipping
Kettlebell	10 curls and 10 rounds
Basketball throws	10 throws and catches of basketball

## 2 Experimental Design, Materials and Methods

### 2.1 Experimental setup

Most of the Human Activity Recognition datasets publicly present [5,6] out there are generated by using either smartphones or smartwatches. Both these devices has a single pair of accelerometer and gyroscope, with 3 axis along -x, -y and -z each. Also, the smartwatches are located on the wrist and the smartphones are usually in kept in the pockets by the chest or waist. Our proposed dataset is collected using a triple synchronous sensors that provides us with reading from 3 different human body points, unlike smartwatches and smartphones that just provides reading from one point. The activities performed by the subjects in the existing datasets are as simple as standing, walking, lying down, etc., whereas on the other hand, the activities we selected for the proposed dataset were more intense and of varying vigor.

**Fig. 1:**
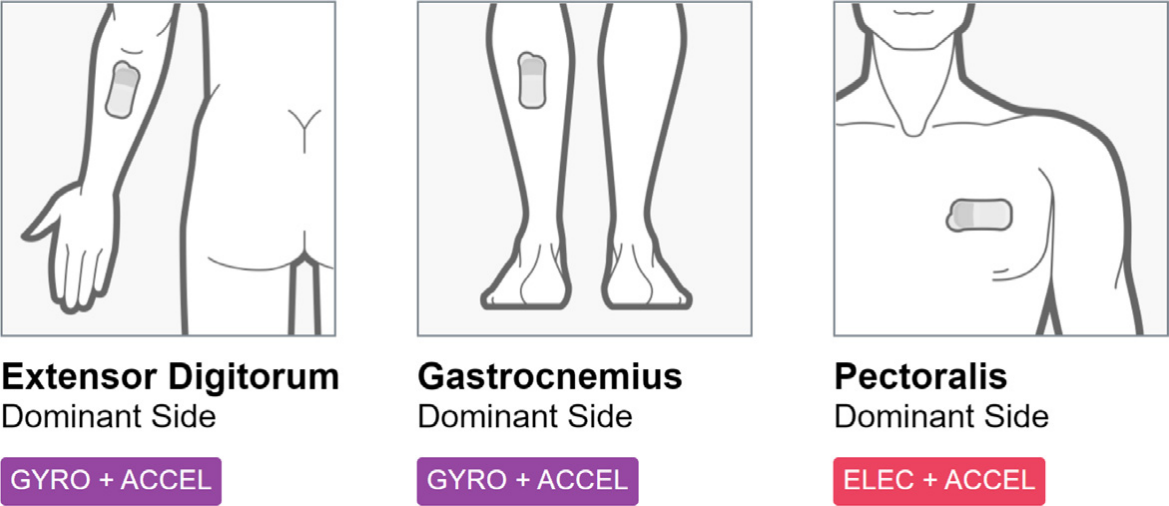
Positioning of the sensors.

This experiment was conducted on 7 healthy adults in the age range of 20 to 40 years, with 4 female and 3 male subjects. Every subject involved in the experiment performed a set of predetermined activities in a supervised environment while wearing the sensors, as shown in Fig. 1.

The sensors used for this experiment were BioStamp nPoint® sensors manufactured by MC10 Inc., a wearable health technology and devices manufacturer. These sensors are externally attached to the skin with the help of the BioStamp adhesive, which contains a gel that amplifies the sensor's electric signals [7]. In our experiment, we made use of three such BioStamp nPoint® sensors. One sensor was attached to the left Pectoralis muscle that gave us the accelerometer and electrode values readings. The other two sensors are attached to the Extensor Digitorum and Gastrocnemius muscles of the subject's dominant side. These two sensors provide the accelerometer and gyroscope values.

Table 7 shows the list of 5 activities performed by all seven subjects along with their description. Each subject was thoroughly supervised, and the start time and time taken to complete each activity in seconds were noted for precision with a stopwatch. Any issues with the activity were purged and the activity was re-performed.

### 2.2 Data cleaning and filtering

We created a python script to filter out the data points that lie outside the time window recorded for every activity using the starting time and elapsed time from every subject's metadata. Using the same metadata, we added the corresponding activity name to each data point to the new activity label column along with the subject ID.

Data obtained from the accelerometer and gyroscope of the sensors attached to the Extensor Digitorum and Gastrocnemius muscles are saved into two different files. Since the frequency of data collection for both, accelerometer and gyroscope is the same for both these sensors, the data points collected are at the exact same time. So, both these files were merged by a simple inner join operation on the timestamp column of the data.

Unlike Extensor Digitorum and Gastrocnemius muscles, the frequency for data collection for accelerometer and electrode values in Pectoralis muscle sensor is different [8]. Due to this, merging these two tables is not as simple as merging accelerometer and gyroscope readings. In this situation, for every data point from the accelerometer reading, we found the closest data point from the electrode values with respect to time. To do this, we subtracted each accelerometer's timestamp value from all the electrode values’ timestamp and selected the data point with the smallest absolute difference, giving us a one-to-one mapping.

We then concatenated the merged tables of each subject together to give us a single table for every sensor (Extensor Digitorum, Gastrocnemius, and Pectoralis). As the final step of data filtering and cleaning, we merged the readings from all three sensors. Extensor Digitorum and Gastrocnemius were merged with an inner join operation, as their frequency is the same. Since the frequency of Pectoralis muscle sensor is different from that of Extensor Digitorum and Gastrocnemius, we performed mapping of Pectoralis sensor values to the data points from Extensor Digitorum and Gastrocnemius by subtracting it's timestamp from Pectoralis timestamp and selecting the data point with the smallest absolute difference, like we did for merging Pectoralis's accelerometer and electrode values.

## Ethics Statement

The data is collected from seven anonymous healthy adult subjects after a valid consent received from all of them. IU IRB: #2010321996

## Declaration of Competing Interest

The authors declare that they have no known competing financial interests or personal relationships which have, or could be perceived to have, influenced the work reported in this article.
